# The Association Between Recent Family Death and Self-Perceptions of Aging Among Middle-Aged and Older Adults

**DOI:** 10.1093/geroni/igaa057.1457

**Published:** 2020-12-16

**Authors:** Shu Xu

**Affiliations:** University of Masschusetts Boston, Dorchester, Massachusetts, United States

## Abstract

The loss of a family member may have a significant influence on one’s aging experience in life. Self-perceptions of aging, which are an individual’s beliefs or evaluation of their experiences of aging, have been described as an important factor for one’s health and daily life. However, there is little research on the association between family death and self-perceptions of aging. This study examines the relationships between recent family death, self-perceptions of aging, and gender of the bereaved among middle-aged and older adults. Using nationally representative data from the Health and Retirement Study (HRS), we conducted cross-sectional analysis on adults age 50 years and older (n=1,839). Self-perceptions of aging were accessed by 8 items derived from the Attitudes Toward Own Aging subscale of the Philadelphia Geriatric Center Morale Scale and the Berlin Aging Study, and we considered recent family death (i.e., parental death, spousal death, sibling death and child death), as well as gender of the bereaved. Multiple linear regression analyses revealed that respondents who experienced recent family death report less positive self-perceptions of aging compared to those who did not experience recent family death (t = 12.40, p < .01). Recent parental death was more negatively related with self-perceptions of aging for bereaved women than for bereaved men (χ2 = 4.28, p < .05). Findings suggest that middle-aged and older adults experiencing recent family loss have less positive self-perceptions of aging, and gender of the bereaved plays an important role in the relationship between parental death and self-perceptions of aging.

